# Ionic liquids for addressing unmet needs in healthcare

**DOI:** 10.1002/btm2.10083

**Published:** 2018-01-19

**Authors:** Christian Agatemor, Kelly N. Ibsen, Eden E. L. Tanner, Samir Mitragotri

**Affiliations:** ^1^ School of Engineering and Applied Sciences Harvard University Cambridge MA 02138

**Keywords:** active pharmaceutical agents, antimicrobial agents, drug delivery, glucose biosensors, ionic liquids, metal‐containing ionic liquids, permeation enhancers

## Abstract

Advances in the field of ionic liquids have opened new applications beyond their traditional use as solvents into other fields especially healthcare. The broad chemical space, rich with structurally diverse ions, and coupled with the flexibility to form complementary ion pairs enables task‐specific optimization at the molecular level to design ionic liquids for envisioned functions. Consequently, ionic liquids now are tailored as innovative solutions to address many problems in medicine. To date, ionic liquids have been designed to promote dissolution of poorly soluble drugs and disrupt physiological barriers to transport drugs to targeted sites. Also, their antimicrobial activity has been demonstrated and could be exploited to prevent and treat infectious diseases. Metal‐containing ionic liquids have also been designed and offer unique features due to incorporation of metals. Here, we review application‐driven investigations of ionic liquids in medicine with respect to current status and future potential.

## 1 INTRODUCTION

**Figure 1:**
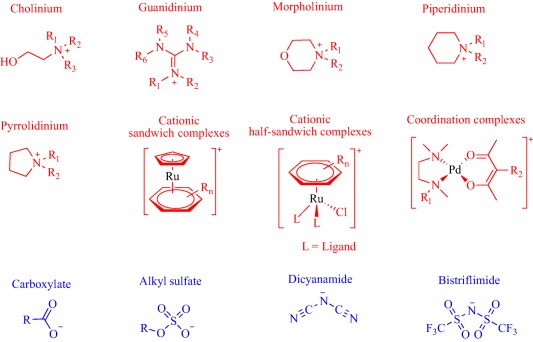
Emerging cationic (red) and anionic (blue) precursors for the design of ILs

Ionic liquids (ILs) have been a topic of scientific interest for over a century,^1^ with one of the earliest reports dating back to 1914 on ethylammonium nitrate by Paul Walden.^2^ It remains one of most active and exciting areas of research with over 7,000 contributions in 2017 alone. The field has benefitted from several distinctive breakthroughs including the report of air‐ and water‐stable imidazolium‐based room‐temperature IL by Wilkes and Zaworotko in 1992,^3^ which challenged previous concepts, and pushed the frontiers of application beyond catalysis, synthetic chemistry and electrochemistry to new landscapes.^4^, ^5^, ^6^, ^7^, ^8^, ^9^, ^10^, ^11^, ^12^, ^13^, ^14^, ^15^, ^16^, ^17^, ^18^ Early advances were motivated to develop ILs as green, nonvolatile, nonflammable, and stable solvents, however, recent findings have broadened the field, redefining ILs as low melting salts (melting point <100°C) with an unlimited suite of tunable properties including toxicity, volatility, flammability, and instability.^15^, ^16^, ^17^, ^18^, ^19^, ^20^, ^21^, ^22^, ^23^, ^24^ The erstwhile narrow perspective that views ILs as salts of quaternary ammonium, imidazolium, pyrrolidinium, pyridinium, or phosphonium cations^15^, ^18^ has broadened as new cations including the bioinspired cholinium and guanidinium cations^25^, ^26^, ^27^, ^28^, ^29^, ^30^, ^31^, ^32^, ^33^, ^34^, ^35^, ^36^, ^37^, ^38^, ^39^, ^40^, ^41^, ^42^, ^43^, ^44^ as well as metal‐containing cations^45^, ^46^, ^47^, ^48^, ^49^, ^50^, ^51^, ^52^, ^53^, ^54^, ^55^ are paired with various anions to afford salts that meet the definition of IL (Figure 1). The evolving complexity of the precursors fosters a conjecture that explains their characteristically low melting point by the structural heterogeneity of a sterically hindered asymmetric cation that impedes strong ionic interaction with the anion as well as precludes ordered packing within a crystal lattice. Also, these recent advances have expanded the chemical space, permitting an unprecedented flexibility in choice of cations and anions to design ILs with tailor‐made properties for fundamental studies and practical applications.

**Figure 2:**
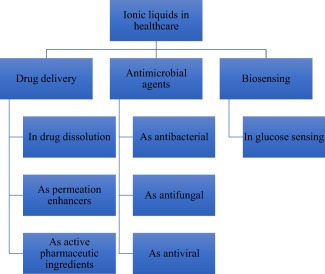
Emerging applications of ILs in healthcare

Application‐driven investigations of ILs is a vibrant and rapidly growing research field with emerging frontiers in healthcare, especially drug delivery (Figure 2). Because many drug candidates exhibit poor solubility profiles, a property that limits their bioavailability, eventually leading to clinical failure, it is expedient to pursue new strategies to solubilize and/or formulate them. An element of this pursuit is the exploration of ILs as drug delivery technologies. In perspective, ILs are liquid salts but with low melting points, typically below 100°C, and in some cases, form an exotic class of room‐temperature solvents that can favorably solvate a wide range of compounds.^18^ Such extraordinary solvating power enables ILs to dissolve many poorly soluble drugs, and ultimately enhances drug permeation through physiological barriers to boost therapeutic efficacy.^6^, ^8^, ^9^, ^10^, ^12^, ^56^ Also, the innovative concept of active pharmaceutical ingredient ionic liquids (API‐ILs) aims at pairing a pharmaceutically active cation and anion to design IL‐based drugs that feature better solubility behavior enabling efficient permeation through various barriers to reach target cells.^6^, ^8^, ^10^, ^12^

Meanwhile, empirical laboratory‐scale results prove the potential of the IL strategy as a promising technology to eliminate several challenges including polymorphism that is associated with some solid‐state drugs, and solubility‐limited bioavailability of poorly soluble drugs.^6^, ^8^, ^10^, ^12^ The ability of ILs to enhance the therapeutic efficacy of drugs is evident from various studies^6^, ^8^, ^10^, ^12^ but clinical exploitation of this technology remains limited. Indeed, only a few API‐ILs, for example the 1‐(2‐hydroxyethyl)pyrrolidinium salts of diclofenac sold as FLECTOR® patch in the United States, have entered the market. This review, therefore, aims to draw the attention of stakeholders in the field of drug delivery to the breakthroughs that unequivocally confirm ILs as technologies that improve the therapeutic efficacy of drugs. To encourage research into metal‐containing ILs, we review this under‐explored subfield and highlight an example of how the synergy between metal‐ and IL‐specific properties enables biosensing application. The antimicrobial activity of ILs is well‐demonstrated, and research in this area is continuing. Indeed, these salts are increasingly explored as antimicrobial, antifungal, and antiviral agents.^29^ Here, we present several examples of ILs with excellent activity against infection‐causing microorganisms. Further, we discuss the central question of toxicity, which must be addressed to translate these innovative materials into marketable healthcare technologies, and recommend future directions to circumvent these problems and expand IL research in healthcare. We are aware of many excellent recent reviews on the biomedical applications of ILs^5^, ^6^, ^7^, ^8^, ^9^, ^10^, ^12^, ^18^ but the prolific research contributions in the field (over 7,000 publications and patents since January 2017) necessitates regular reviews and perspectives.

### 1.1 Emerging opportunities in drug delivery

The flexibility to fine‐tune the physicochemical properties of ILs provides effective solutions to many drug delivery challenges such as low bioavailability and drug insolubility. In this section, we highlight how ILs are being positioned as permeation enhancers, solubility promoters, and API‐ILs.

#### 1.1.1 Enhancing cell membrane permeability

Drug delivery systems are critical determinants of a drug's efficacy and must be designed to protect the drug from undesirable metabolism, and to transport the drug to its target at a desirable rate.^57^ However, various physiological hurdles exist that hinder the efficient transport of drugs. For instance, transdermal and topical drug delivery, promising alternatives to the oral and injection routes, remains challenged by the impervious stratum corneum (SC), which poses a formidable barrier to absorption of chemicals. Many technologies including chemical enhancers are, therefore, deployed to disrupt these barriers to ensure efficient drug transport.^58^, ^59^, ^60^, ^61^, ^62^, ^63^, ^64^, ^65^, ^66^, ^67^, ^68^, ^69^, ^70^, ^71^, ^72^, ^73^ ILs have attracted attention in this regard, and are now increasingly investigated as chemical enhancers to perturb cell membranes with the goal of improving transcellular and paracellular drug transport.^67^ Indeed, computational models, as well as experimental data, prove the feasibility to enhance drug transport using ILs. For instance, molecular dynamics coupled with an empirical force field showed that the cationic head of the amphiphilic 1‐octyl‐3‐methylimidazolium‐based IL inserts into a model cell membrane, disrupting structural integrity to increase the membrane permeability of small polar molecules such as ammonia.^74^ Empirical data confirmed that hydrophilic imidazolium‐based ILs fluidize the cell membrane to create pathways for the diffusion of molecules.^75^ Although these findings linked the IL‐enhanced permeability of biological membranes with cytotoxicity, it is plausible to assume that these mechanisms should enable transport of drugs to target sites. Also, the ability of ILs to extract lipids from physiological structures such as the SC is now recognized.^30^ Indeed, such lipid‐targeted effects creates defects that enhance drug permeation. Physicochemical properties permitting, different IL‐based enhancers operate in distinct mechanistic fashion to compromise structural and functional integrity of physiological barriers and enable efficient drug trafficking. Hydrophilicity, for instance, opens the tight junction to favor paracellular transport while hydrophobicity preferentially enables partitioning into the epithelial membrane to enhance transcellular transport.^72^ Again, depending on concentration, polar enhancers preferentially interact and insert within the protein or lipid regions of the SC causing fluidization, whereas nonpolar enhancers mainly target the lipid regions to eliminate barriers, creating channels for the diffusion of molecules.^76^

**Figure 3:**
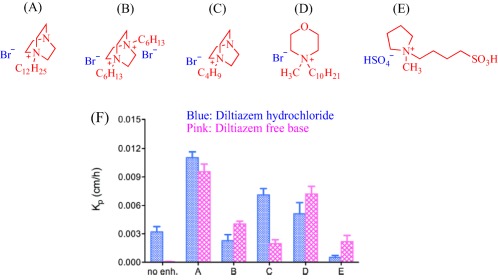
IL enhances permeability of diltiazem hydrochloride into excised rat skin (*K*p, permeability coefficient). Adapted from “Ionic Liquids as Potential Enhancers for Transdermal Drug Delivery,” by D. Monti et al., 2017, International Journal of Pharmceutics, 516, pp. 45–51

As an alternative to aromatic pyridinium and imidazolium cations, alicyclic cations are intensively investigated with the goal of accessing new properties. Monti et al. explored alicyclic pyrrolidinium‐, morpholinium‐, and 1,4‐diazabicyclo[2.2.2]octane‐based ILs as transdermal enhancers for the delivery of diltiazem, a nondihydropyridine calcium channel blocker that treats hypertension^67^ (Figure 3). The results confirmed that the nature of the drug and the IL dictates the degree of transdermal permeation. For example, monocationic 1,4‐diazabicyclo[2.2.2]octane outperforms the ineffective dicationic congener by over twofold in the delivery of diltiazem hydrochloride salt.^67^ Also, morpholinium‐based IL enhances the permeation of diltiazem hydrochloride salt into the skin better than pyrrolidinium‐based IL, which acts as a retarder under this scenario. When administered as a free base, diltiazem permeability through the skin remarkably increases and the dicationic 1,4‐diazabicyclo[2.2.2]octane and the pyrrolidinium‐based ILs function as enhancers.

**Figure 4:**
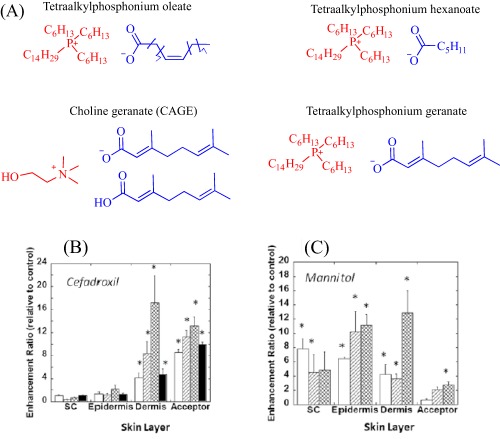
ILs (a) enhances drug (b and c) permeation into excised porcine skin (open bar: tetraalkylphosphonium oleate; hatched bar: tetraalkylphosphonium hexanoate; cross‐hatched bar: choline geranate; closed bar: tetraalkylphosphonium geranate). Adapted from “Ionic Liquids as a Class of Materials for Transdermal Delivery and Pathogen Neutralization,” by M. Zakrewsky et al., 2014, Proceedings of National Academy of Sciences of the United States of America, 111, pp. 13313–13318

The intrinsic toxicity of some IL constructs inspires the exploration of biomolecules as leads to design the next‐generation of ILs. An emerging bioinspired lead molecule is the cholinium cation, present in the cell membrane as a component of phosphatidylcholine and sphingomyelin, and proven to penetrate the cell membrane efficiently.^42^ By pairing a cholinium cation with two equivalents of geranic acid—a biomolecule that functions as a pheromone in some insects^77^ and as a tyrosinase inhibitor in lemongrass^78^—we developed choline geranate (CAGE), a type 3 deep eutectic solvent (DES), for drug delivery^29^, ^30^, ^31^ (Figure 4). DESs share many general physical properties, such as high viscosity and low vapor pressure, with ILs but are distinct by their facile synthesis and the better‐understood toxicity of their precursor.^43^ Therefore, for simplicity, this review discusses CAGE and other DESs under ILs. CAGE is a dual‐functional technology, acting as a broad‐spectrum antimicrobial agent and as a chemical enhancer for transdermal delivery.^31^ Compared to controls, the drug delivery properties of CAGE is better, being able to enhance the delivery of mannitol, a model hydrophilic drug, and cefadroxil, a model antibiotic, by 5‐ and 16‐fold increases, respectively.^31^ We designed other ILs such as choline oleate, tetraalkylphosphonium oleate, tetraalkylphosphonium hexanoate, and tetraalkylphosphonium geranate that act as efficient permeation enhancers, enabling a fivefold enhancement in the transport of cefadroxil into the dermis^31^ (Figure 4).

Enhancement depends, among other factors, on the structure and chemistry of the IL, and therefore, is not a generic property of cholinium‐based ILs. Indeed, choline urea and choline hexanoate, classic examples of DESs, retard the transdermal delivery of mannitol.^31^ To expand the scope of application of CAGE, our group explored its potential to transdermally deliver high molecular weight macromolecules including bovine serum albumin, ovalbumin, and insulin.^30^ In these cases, CAGE outperformed conventional chemical permeation enhancers such as ethanol and Transcutol®, efficiently driving the penetration of fluorescein isothiocyanate‐labeled BSA, OVA and insulin better than the conventional enhancers. The excellent transdermal insulin delivery property of CAGE is observable in male Wister rats as well, where a 25% decrease in blood glucose level occurred 2 hr after applying 10 U ml^−1^ insulin‐CAGE mixture to the skin.^30^

Like CAGE, other liquid salts derived from aliphatic amines and carboxylic acid enhance the permeability of drugs through physiological barriers. The Kubota group recently reported on the mechanism of action of these class of ILs by investigating a series designed by reacting octanoic or isostearic acid with diisopropanolamine or triisopropanolamine.^79^ An equimolar mixture of aliphatic acid and amine yields an IL that co‐exists with unreacted carboxylic acid and amine. Presumably, the unreacted acid and amine contribute to the permeability‐enhancing property of these ILs, which prove excellent enhancers for hydrophilic drugs, modeled by phenol red, but retarders for hydrophobic drugs, exemplified by tulobuterol.^79^ This behavior contrasts with that of the well‐recognized chemical enhancer, Azone, that efficiently transports tulobuterol through the skin but is ineffective with phenol red,^79^ indicating a mechanism of action that is distinct from that of the ILs. This finding led Kubota et al. to hypothesize that ILs derived from aliphatic acids and amines self‐assemble into nanoparticles with the polar head groups forming a hydrophilic core, and the alkyl chain constituting the hydrophobic corona.^79^ Their proposed model explains the unique enhancing property of the ILs. Descriptively, the hydrophilic core more favorably encapsulates the hydrophilic phenol red than the hydrophobic tulobuterol while the hydrophobic corona acts as a permeant, carrying the encapsulated drug through the hydrophobic SC. Although, formation of nanoparticles was not directly confirmed, the self‐assembling of ILs into nanostructures is well‐demonstrated^24^ and, therefore, supports the hypothesis.

#### 1.1.2 Assisting dissolution of poorly soluble drugs

ILs feature excellent solvating power to assist the dissolution of sparingly soluble drugs, leading to improved absorption, and eventually better bioavailability. A classic example is the IL‐assisted transdermal delivery of acyclovir, an antiviral drug with poor bioavailability (15–30%) due to poor solubility (0.05% wt) in water.^80^, ^81^ Goto and coworkers tackled this challenge, increasing the solubility (>10%) by dissolving acyclovir in hydrophilic ILs with strong hydrogen bond acceptors such as dimethylimidazolium dimethylphosphate.^80^, ^81^ Because the hydrophobic SC poses a barrier to the hydrophilic IL, Goto and coworkers obtained zero drug diffusion through the skin. However, they achieved diffusion by developing an IL‐in‐oil (IL/oil) microemulsion, in which the IL phase encapsulates acyclovir while the continuous oil phase permeates the hydrophobic barrier to deliver the cargo. Franz‐type diffusion cell experiments showed that the IL/oil microemulsion allows the delivery of acyclovir into Yucatan hairless pig skin.^80^ Also, an IL‐in‐water (IL/water) microemulsion enhances the permeation of poorly water‐soluble drugs as demonstrated with the ex vivo topical delivery of etodolac, a nonsteroidal anti‐inflammatory drug, to rats.^82^ The IL/water microemulsion formulation permits the efficient transport of etodolac into the skin, resulting in a comparatively more effective therapeutic performance compared to formulations without ILs. In a recent development, the Goto group applied a 1‐dodecyl‐3‐methylimidazolium‐based IL to enhance the skin permeability of a solid‐in‐oil nanodispersion to deliver ovalbumin in vitro.^83^

**Figure 5:**
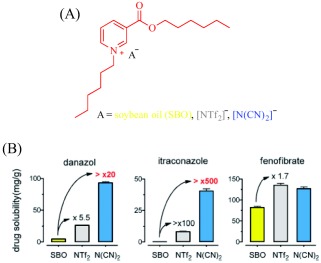
ILs (a) promote dissolution of poorly soluble drugs—danazol, and itraconazole (b). Adapted from “Ionic Liquids Provide Unique Opportunities for Oral Drug Delivery: Structure Optimization and In Vivo Evidence of Utility,” by H. D. Williams et al., 2014, Chemical Communications, 50, pp. 1688–1690

The potential of IL‐assisted drug solubilization and permeation extends beyond transdermal and topical routes of drug administration as proven successes are evident from studies on oral routes as well. For instance, Porter and coworkers enhanced oral delivery of poorly water‐soluble drugs such as danazol and itraconazole by exploiting the solvating power of ILs. By changing the length of the alkyl substituents and the counteranion of a 1‐hexyl‐3‐hexyloxycarbonylpyridinium‐based IL, they tuned the hydrophobic/hydrophilic balance to control the solubility of these drugs.^84^ Replacing the hydrophobic anion, bis(trifluoromethane)sulfonimide ([NTf_2_]^–^), with a hydrophilic anion, dicyanamide ([N(CN)_2_]^–^), results in a 3.6‐ and 5‐fold increase in the solubility of danazol and itraconazole, respectively (Figure 5). With judicious selection of the anion, it is possible to combine the hydrogen bonding properties of [N(CN)_2_]^–^ with the hydrophobicity of [NTf_2_]^–^ in one platform to achieve a solubility profile that permits dissolution of poorly water‐soluble drugs. For instance, alkyl sulfate anions are hydrogen bonding and hydrophobic and, therefore, enable dissolution of danazol to an extent that competes with [N(CN)_2_]^–^. It is worth noting that these 1‐hexyl‐3‐hexyloxycarbonylpyridinium‐based ILs feature little advantages for the delivery of lipid‐soluble drugs such as fenofibrate over the classical excipients because enhancement in solubility is marginal.^84^ When formulated into a self‐emulsifying drug delivery system (SEDDS), all the ILs but the [N(CN)_2_]^–^‐containing IL, maintain the solubilized danazol in simulated gastrointestinal fluid but lacks an advantage over lipid‐based formulations in ensuring drug absorption in rats.^84^ The SEDDS derived from [NTf_2_]^–^ or [C_10_SO_4_]^–^ gives lower danazol plasma concentration while that from [C_18_SO_4_]^–^ is comparable with the lipid‐based system due to lipids‐assisted absorption of the encapsulated drug.^84^

#### 1.1.3 Designing active pharmaceutical ingredients‐ionic liquids

An estimated 40–70% of promising drug candidates fail because of solubility‐limited bioavailability.^12^, ^85^ Consequently, the pharmaceutical industry is actively exploring several strategies to improve drug dissolution as well as improve other pharmacological properties that ensure therapeutic efficacy. The concept of API‐IL is an innovative solution to address the inherent problems of many drug candidates, with the overall goal of realizing better efficacy. Rogers and coworkers pioneered this concept, rationally combining discrete cationic and anionic derivatives of approved drugs to control various pharmaceutical cocktail properties including drug release, stability, solubility, and bioavailability.^7^, ^12^, ^86^, ^87^, ^88^ For example, by pairing lidocaine, a widely used anesthetic for postsurgical and neuropathic pain, with sodium docusate, a laxative, the Rogers group obtained lidocaine docusate, which cost‐effectively delivers its constituent drugs with improved pharmacological properties and a possibility for new bioactive properties.^88^ Specifically, the antinociceptive effect of lidocaine docusate is superior to that of lidocaine as evidenced by the improved and more extended therapeutic impact in a mouse model.^88^ The API‐IL approach alters the mechanism of action of lidocaine at the cellular level. Lidocaine docusate exerts an effect that is distinct from that of lidocaine on neuritic outgrowth in pheochromocytoma (PC12) cells.^88^ Also, the API‐IL approach was used to design ranitidine docusate, from ranitidine hydrochloride and sodium docusate.^88^ The successful design of liquid ranitidine docusate eliminates polymorphism inherent in ranitidine.

The pioneering work of Rogers and coworkers on molecular engineering of approved pharmaceutically active molecules into API‐ILs to access improved pharmacological fingerprints opens new opportunities in pharmaceutics. The ability to facilely obtain and characterize ILs fosters the growing interest in the field as evidenced by the snowballing repository of API‐ILs (Table 1). Indeed, numerous successful explorations of the concept to enhance the property of approved drugs exists in the literature and are illustrated here within the context of selected examples. A classic example is the pairing of the cations—cholinium, tetrabutylphosphonium, tetrabutylammonium, or tetramethylhexadecylammonium—or anions—chloride or docusate—with the appropriate complementary acyclovir ion to achieve over two‐order of magnitude enhancement in the aqueous solubility of acyclovir.^86^ Compared with the parent acyclovir, the API‐ILs derived from acyclovir anions exhibit better solubility in water with even the hydrophobic tetrabutylphosphonium counterion giving 200 times improvement in solubility.^86^ Also, but to a lesser degree, pairing the acyclovir cation with a chloride or docusate anion gives API‐ILs with a better aqueous solubility profile than the neutral acyclovir but no advantage over the sodium salt of acyclovir, whose solubility is superior to that of the docusate anion and comparable to the chloride anion.^86^ The excellent solubility of the acyclovir API‐IL is evident in simulated gastric and intestinal fluid, where the cholinium‐based API‐IL, for instance, is 650 times more soluble in the intestinal fluid than the parent drug.^86^

**Table 1:** Demonstrated advantages of API‐IL over parent drugs

API‐IL^a^		
Cation	Anion	Advantages over parent drug
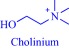	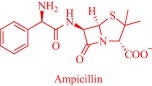	Broader solubility spectrum.^89^
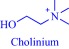	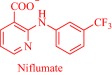	Enhanced hydrophilicity without increased in cytotoxicity.^34^
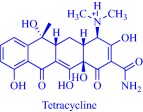	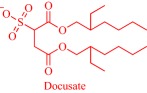	Enhanced lipophilicity and membrane permeability.^90^
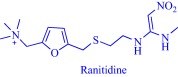	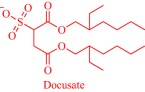	Absence of polymorphism.^88^
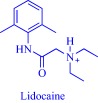	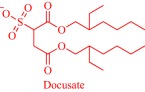	Enhanced lipophilicity as well as enhanced and extended therapeutic effect.^88^
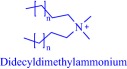	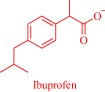	Dual functionality, exhibiting antibacterial and anti‐inflammatory properties.^88^
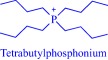	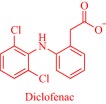	Faster dissolution rate in phosphate buffered saline (PBS).^91^
	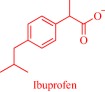	
	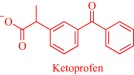	
	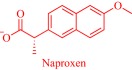	
	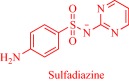	
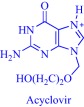	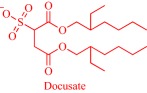	Improved hydrophobicity resulting in better drug delivery capacity.^86^
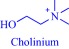	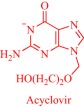	Enhanced hydrophilicity, specifically 450 and 650 times improved solubility in PBS in simulated intestinal fluid.^86^
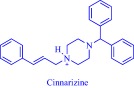	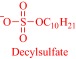	Enables higher dosage into SEDDS.^92^
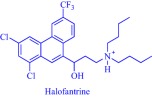	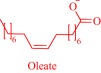	Enhanced lipophilicity.^92^
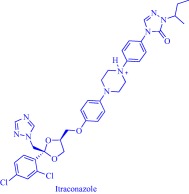	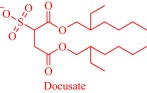	Enables higher dosage into SEDDS.^92^
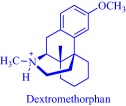	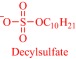	Enhanced solubility leading to higher dosage into SEDDS.^92^
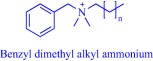	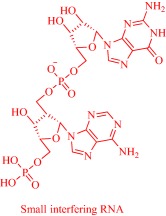	Enhanced siRNA transdermal transport.^93^

^a^The cation and anion are paired to obtain an API‐IL.

Also, by coupling cholinium with pharmaceutically active anions, the Marrucho group advanced the physicochemical and pharmaceutical properties of the anions.^34^, ^90^, ^94^ Specifically, API‐ILs derived by combining cholinium with anions of nalidixic acid, niflumic acid, 4‐aminosalicylic acid, pyrazinoic acid, or picolinic acid exhibit better solubility in water, gastric, and intestinal fluid.^34^ Enhanced solubility is presumed to boost membrane permeability, increasing bioavailability and eventually therapeutic efficacy but could lead to over‐dosage that is detrimental to critical cellular processes. However, the cholinium, nalidixic, niflumic, and pyrazinoic API‐ILs feature similar cytotoxicity toward Caco‐2 colon carcinoma cells and HepG2 hepatocellular carcinoma cells as the parent drugs despite the superior solubility of the API‐ILs.^34^

It may be imperative that a drug penetrates the cell membrane to perform its therapeutic functions. Several parameters, extrinsic and intrinsic to the drug, control the dynamics of cell membrane permeability with the hydrophobic/hydrophilic balance being a key intrinsic factor.^95^ A rational design strategy offers the possibility to modulate the balance by an appropriate combination of an ionic drug with a complementary ion. Exemplarily, the Marrucho group tuned the hydrophobic/hydrophilic balance of L‐ampicillin by coupling its anion to ammonium, phosphonium, pyridinium, or imidazolium cations.^94^ Compared to the parent drug and its sodium salt, the API‐ILs featured high affinity for the cell membrane as revealed by their higher octanol–water partition coefficient,^94^ a parameter that describes drugs' lipophilicity.^95^ Indeed, lipophilicity is a determinant of how drugs partition into the lipid bilayer, and how they are distributed and metabolized in the body.^95^ Marrucho and coworkers manipulated the hydrophobic/hydrophilic balance by molecularly engineering the polarity of the cationic head group to tune hydrophilicity.^94^ Counterintuitively, the increased hydrophilicity of the ampicillin‐based API‐ILs did not compromise the octanol–water partition coefficient. Even the sodium salt of ampicillin with a similar aqueous solubility profile as the cholinium‐based API‐IL, for instance, is less lipophilic,^94^ suggesting the superiority of the API‐IL approach to the traditional salt formation strategy of controlling solubility in the pharmaceutical industry. Evidently, the API‐IL approach is a more versatile strategy that allows synchronized control of various determinants of drugs' efficacy such as solubility, hydrophobic/hydrophilic balance, drug absorption into systemic circulation and biocompatibility.

Solubility is central to drug absorption, bioavailability, and eventually therapeutic efficacy. The API‐IL approach is a powerful strategy to promote drug absorption and bioavailability by transforming poorly water‐soluble drugs into lipophilic API‐ILs, which are more efficiently absorbed from the intestinal fluid than the parent drug. Porter and coworkers exploited this strategy to improve the bioavailability of itraconazole, cinnarizine, and halofantrine, archetypes of poorly water‐soluble drugs with low bioavailability, by pairing with alkyl sulfate, carboxylate or [NTf_2_] anion.^92^ The lipophilicity of the resultant API‐ILs ensures their formulation into SEDDS and maintains their solubility in simulated intestinal fluid. Compared with control formulations, oral administration of the SEDDS to rats gives a 2‐ and 20‐fold increase in cinnarizine and itraconazole plasma concentration, respectively.^92^ Recently, our group used the API‐IL strategy to simultaneously tune physicochemical properties, skin permeability, absorption, and biocompatibility of small interfering RNA (siRNA) by pairing it with a benzyl dimethyl alkyl ammonium cation.^93^ The strategy upgraded the hydrophobic/hydrophilic balance of the siRNA, transforming the hydrophilic neat siRNA to a lipophilic siRNA‐based API‐IL. Increased lipophilicity is expected to enhance skin permeability, and absorption of siRNA. Indeed, Franz diffusion cells and confocal laser scanning microscope experiments unequivocally confirm enhancement of these properties. To illustrate, upon transformation to API‐IL, the percentage of siRNA delivered to the viable epidermis of pig skin increases from 2.06 ± 0.15 to 9.85 ± 2.64%, and internalization of siRNA by human adult epidermal keratinocytes (HEKa) improves.^93^ Changing the octyl substituent on the benzyl dimethyl alkyl ammonium cationic component of the API‐IL to tetradecyl enhances the percent delivery. Also, the nature of the alkyl group influences biocompatibility because replacing the octyl with a tetradecyl or stearyl group increases cytotoxicity toward HEKa. Neat siRNA is more biocompatible to HEKa than the API‐ILs, a finding that concurs with the established positive correlation of hydrophobicity with cytotoxicity. Nonetheless, the API‐IL platforms more effectively treat skin diseases than the neat RNA,^93^ which is an advantage.

### 1.2 Incorporating metals in ionic liquids to sense biomolecules

Incorporating a metal center into an organic molecule integrates the unique magnetic, photo‐activity, and redox‐activity of metals with the inherent stability and processibility of organic molecules.^96^ Toward this direction, metal‐containing ILs are emerging as counterparts of purely organic‐based ILs with the intent of imparting new functions to liquid salts.^45^, ^46^, ^47^, ^48^, ^49^, ^50^, ^51^, ^52^, ^53^, ^54^, ^55^ This development challenges the concept of ILs as organics salts as the realm opens to organometallic and coordination chemistry. Progress in this emerging field is slow due to challenges inherent in the synthesis of organometallic and coordination compounds but is exciting and promising as some exotic properties such as magnetism and luminescence are attainable. Some reports on the synthesis of metal‐containing ILs exist in the literature, but knowledge about their applications is scarce. However, recently, Senthilkumar and coworkers reported a cobalt (Co)‐containing salophen‐type IL for the nonenzymatic sensing of glucose.^45^ Glucose sensing forms an integral element in the management of diabetes mellitus, a metabolic disorder that kills 1.5 million people in 2012 worldwide because of elevated blood glucose level that causes life‐threatening issues.^97^ In contrast to enzyme‐based glucose sensors, the metal‐based nonenzymatic technology is cheap, robust, and easy to fabricate, making it an attractive option in the quest for new glucose biosensors.^45^ The ability to tune the redox activity of a transition metal via selection of an appropriate ligand endows this emerging technology with a flexibility that is absent in the enzyme‐based technology. Incorporating metals into ILs, therefore, should trigger a synergy between physicochemical properties, such as ionic conductivity, of the IL and the redox activity of the metal as well as synchronously modulate these properties.

**Figure 6:**
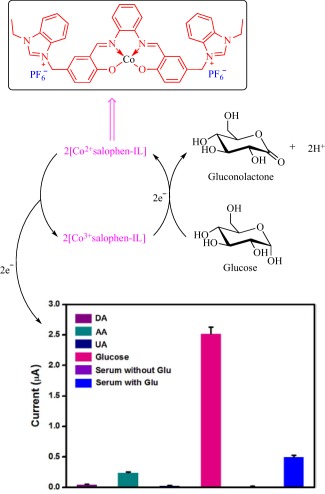
Schematic depicting the chemistry of Co‐containing IL in the detection of glucose (DA, dopamine; UA, uric acid; AA, ascorbic acid; Glu, glucose). Adapted from “A Bioinspired Ionic Liquid Tagged Cobalt‐Salophen Complex for Nonenzymatic Detection of Glucose,” by M. Benjamin et al., 2017, Biosensors and Bioelectronics, 91, pp. 380–387

Senthilkumar and coworkers fabricated the metal‐containing IL‐based glucose sensor by immobilizing a Co‐containing salophen IL (salophen IL is *N*,*N*′‐(o‐phenylene)bis‐[(3‐ethyl‐1*H*‐benzimidazole‐1‐ium‐1‐yl)methylene hexafluorophosphato]salicylideneimine) (Figure 6) on electrochemically reduced graphene oxide deposited onto a screen‐printed carbon electrode.^45^ Compared to an unmodified electrode in the presence of glucose, the modified electrode gives higher anodic current with an onset that corresponds to Co^2+/3+^ redox couple. Mechanistically, glucose sensing occurs as Co^2+^ electrochemically oxidizes to Co^3+^, which then oxidizes glucose to gluconolactone while being reduced to Co^2+^. The ionically conducting IL permits efficient transfer of electrons to drive a redox cycle if glucose is present. Senthilkumar and coworkers demonstrated the ability of the IL‐modified electrode to sense glucose in real human blood serum and urine with the detected concentration concurring with the certified values^45^ (Figure 6).

### 1.3 Fighting microbial infections

Microbial infections continue to challenge scientific achievements, drain global resources, and threaten human survival. The global burden of infectious disease is worrying as revealed by the World Health Organization (WHO) 2017 health statistics that estimated 429,000 deaths from malaria, 1.1 million from HIV‐related illnesses, 1.3 from hepatitis, and 1.4 million from tuberculosis in 2015.^98^ The same year, 1.6 billion people sought treatment for neglected tropical diseases globally, another indicator of the enormous burden of microbial infections.^98^ The prevalence of antimicrobial resistance worsens the already bleak scenario, stressing the need for an urgent action plan to tackle the menace of infectious diseases. An element of the WHO action plan advocates development of new antimicrobial agents^99^ and in this regard, scientists are pursuing new strategies to replenish the exhausted pipeline with efficacious antimicrobial agents. A recent strategy is the exploration of ILs as a platform to prevent and treat infections.

Inspired by the well‐established antimicrobial property of cationic molecules, such as quaternary ammonium compounds, as well as the eco‐toxicity of ILs, scientists are investigating their antimicrobial activity.^100^ The IL strategy modulates the antimicrobial activity of the cationic head group via rational selection of the counteranion. For instance, the antimicrobial activity of the salt, benzalkonium chloride, is tuned by transformation into an IL as shown in the activity of benzalkonium saccharinate and benzalkonium acesulfamate against *Streptococcus mutans*, *Staphylococcus epidermidis*, and *Candida albicans*.^87^ Compared to the chloride, which exhibits a minimum bactericidal concentration (MBC) value of 16 ppm against these microorganisms, benzalkonium saccharinate is more potent against *S. mutans* (MBC = 0.5 ppm) but less potent against *S. epidermidis* (MBC = 31.2 ppm) and *C. albicans* (MBC = 31.2 ppm). The substituents on the cationic head group play a profound role in the overall activity; for example, dodecyldimethylammonium saccharinate and acesulfamate with a quaternary ammonium head group are distinct in activity from their benzalkonium congeners.^87^

Cholinium‐based ILs are quaternary ammonium‐based salts, and therefore, are expected to exhibit antimicrobial activity. Cholinium‐based ILs are potent antimicrobial agents, acting against diverse microbial life forms including viruses, bacteria, fungi, and algae. Depending on the anion, microorganism, and substituent on the cationic head group, they could be more potent than well‐recognized toxic compounds such as atrazine, benzene, and chloroform.^33^ This is exemplified by the superior activity of cholinium dihydrogenocitrate over atrazine and benzene in inhibiting the bioluminescence of *Vibrio fischeri*.^33^ Also, the activity of cholinium dihydrogenocitrate against this microorganism exceeds that of some imidazolium‐, pyridinium‐, and pyrrolidinium‐based ILs, stressing the importance of molecular structure on toxicity.^33^ A positive correlation between activity and anion hydrophobicity suggest permeation through the cell membrane is an important culprit in toxicity against *V. fischeri*.^33^ Such a correlation is also evident in the activity of a series of cholinium alkanoates‐based ILs against the fungi, *Penicillium brevicompactum*, *P. glandicola*, and *P. corylophilum*.^25^ In the case of *V. fischeri*, the hydrophobicity of the substituent on the cationic head group as well as the presence of a polar functional group on the substituent is a less important determinant of activity, contradicting the established role of these parameters on toxicity.^33^ A complete mechanistic framework for bioactivity of quaternary ammonium‐based ILs, therefore, includes membrane disruption and permeation activated by a complex interplay of structural features including anion, substituent, and the cationic head group. Indeed, the nature of anion, cation, and substituent influences the degree of permeation.

**Figure 7:**
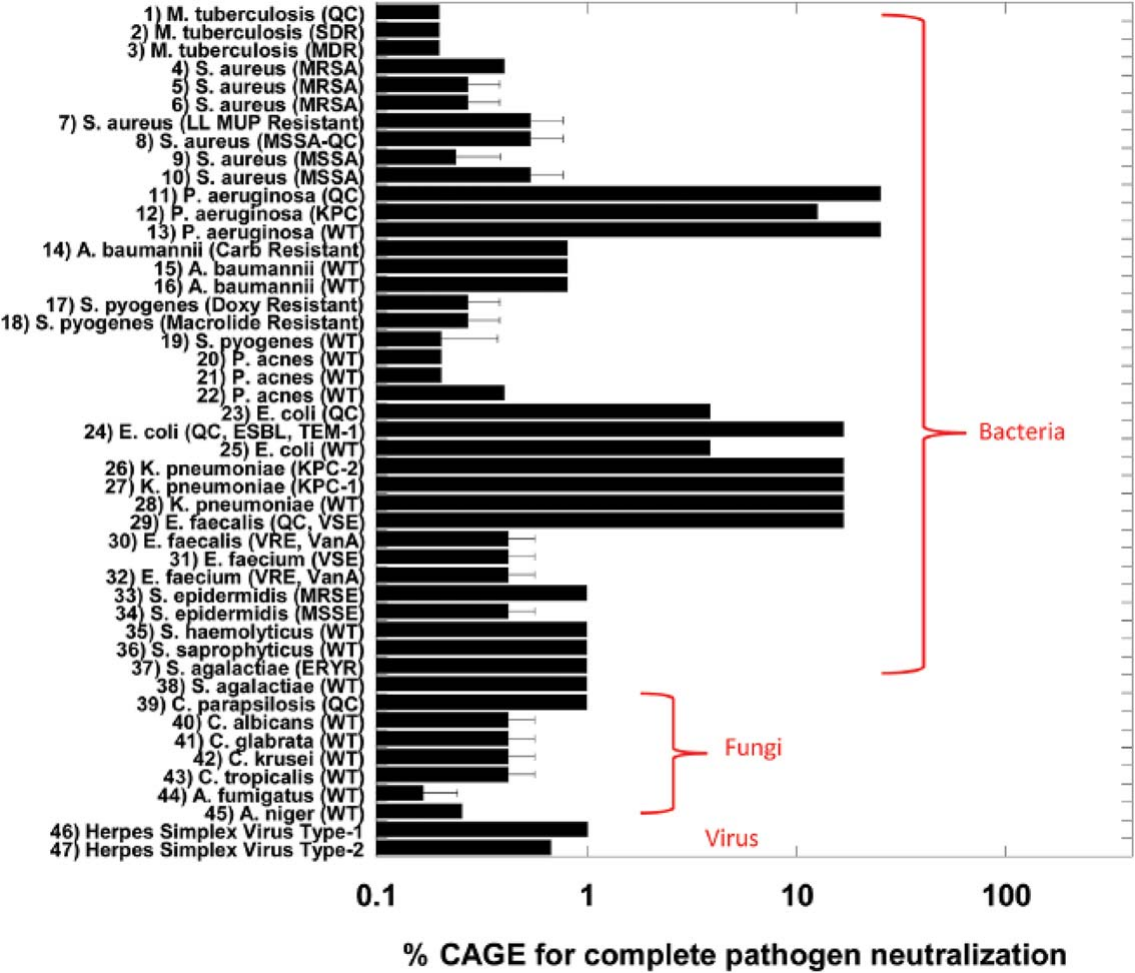
CAGE (choline geranate [for structure see Figure 2]) is a potent broad‐spectrum antimicrobial agent. Adapted from “Choline and Geranate Deep Eutectic Solvent as a Broad‐Spectrum Antiseptic Agent for Preventive and Therapeutic Applications,” by M. Zakrewsky et al., 2016, Advances Healthcare Materials, 5, pp. 1282–1289

A hydrophobic anion bolsters the activity of cholinium‐based ILs against *V. fischeri* 33 but a hydrophilic anion such as an amino acid‐derived anion undermines activity against *Escherichia coli*, *Staphylococcus aureus*, *Salmonella enteritidis*, and *Listeria monocytogenes*.^32^ Activity increases with lipophilicity of amino acid‐derived anions and upon insertion of a hydroxyl group on the anion but decreases with the addition of a carboxyl group.^32^ The use of a highly lipophilic anion such as geranate as in CAGE results in potent activity against biofilm Gram‐negative *Pseudomonas aeruginosa* and *Salmonella enterica*. Other cholinium‐based ILs exemplified by choline malonate and choline hexanoate effectively eradicate 72‐hr *S. enterica* biofilm while benzethonium chloride, tetraakylphosphonium hexanoate, and tetraakylphosphonium geranate reduce 72‐hr *P. aeruginosa* biofilm by ∼5 log_10._ 31 Activity of the ILs depends on the duration of growth with a biofilm being less susceptible with increase in time. CAGE is also effective against a diverse range of microorganisms including viruses, bacteria, fungi and, more importantly, drug‐resistant and resilience strains such as methicillin‐resistant *S. aureus*, *Acinetobacter baumannii*, *Mycobacterium tuberculosis*, *Candida albicans*, and Herpes Simplex Virus Type‐2, which are eradicated by less than 1% CAGE^29^ (Figure 7). The mechanistic pathway leading to the potent activity of CAGE remains elusive but evidence from dynamic light scattering overruled physical destruction with delipidation or fluidization of membrane being more likely.^29^

Apart from ILs derived from a quaternary ammonium cationic head group, other head groups exert excellent activity against microorganisms. Alkylimidazolium‐ and alkylpyridinium‐based ILs are active against bacteria including *E. coli*, *S. aureus*, *Bacillus subtilis*, *Pseudomonas fluorescens*, and *Saccharomyces cerevisiae* with activity increasing with length of alkyl chain but unaffected by the nature of the anion.^101^ Similarly, the activity of alkylimidazolium‐based ILs against algae such as *Scenedesmus obliquus* and *Chlorella ellipsoidea* increases with the length of alkyl chain^102^ whereas that against *Scenedesmus vacuolatus* decreases with the use of a dicationic head group.^103^

The antimicrobial activity of ILs has been explored at the macromolecular level with poly(ionic liquid)s as well. Yan and coworkers reported poly(ionic liquid)‐based membranes for antimicrobial wound dressings developed from quaternary ammonium, imidazolium, or pyrrolidinium, and in some cases, incorporated metals such as Zn, Cu, or Fe into the poly(ionic liquid)s.^104^, ^105^, ^106^ These polymeric ILs exhibit higher antibacterial activity against *E. coli* and *S. aureus* than the corresponding nonpolymeric ILs, and the presence of metal center enhances activity.^104^, ^105^, ^106^

### 1.4 Toxicity of ionic liquids

To achieve clinical relevance and secure regulatory approval, the therapeutic benefit of a drug should significantly offset known risks. In contrast to organic solvents, ILs are typically nonvolatile and nonflammable, which led to the concept of “ILs as green chemicals.” Obviously, substituting a volatile organic solvent with a nonvolatile IL in a chemical or biochemical process decreases inhalation exposure, flammability hazard, and environmental pollution. Regardless, to categorize ILs, as a class, intrinsically safe and green is premature until a central element of toxicity, biocompatibility, has been established. It is, therefore, pertinent that researchers exploring ILs for biomedical applications establish the biocompatibility profiles.

Compelling empirical data prove the toxicity of many ILs toward diverse life forms, ranging from nucleic acids to multicellular organisms (Table 2). Various in silico^74^, ^75^, ^113^, ^114^, ^115^, ^116^, ^117^ as well as in vivo and in vitro (Table 2) studies have unraveled the intricacies and molecular origin of this toxicity and enriched our understanding of the critical parameters that control toxicity. Most studies examining toxicity employed in silico or in vitro approaches and a majority of the small number of in vivo studies uses nonmammalian animal models. Regardless of this limitation, it is now evident that toxicity is tunable and depends on the anion, cation, concentration, substituents, and the investigated biosystem.^13^, ^15^, ^16^, ^17^, ^18^, ^22^ The effect of the cationic head group is complicated with a clear trend difficult to establish due to the structural diversity of ILs. Nonetheless, the cationic charge density influences the extent of toxicity as evidenced by the apparent difference in toxicity of tetrabutylphosphonium (Log IC_50_ ∼ 2.58–2.64 μM) and tetrabutylammonium (Log IC_50_ ∼ 2.25–2.35 μM) cationic head groups in inhibiting the activity of electric eel acetylcholinesterase.^118^ Compared to nitrogen, the relatively larger atomic radius of phosphorus decreases the cationic charge density of the phosphonium head group, eventually weakening electrostatic interactions with endogenous anionic biomolecules. Of course, such interactions play a pivotal role in the toxicity of cationic bioactive molecules including ILs.^119^ In principle, therefore, modulating the charge density via substitutions on the cationic head group may offer a strategy to tune the toxicity profiles of ILs. But in practice, it is complex due to the interplay of other critical parameters such as side chain polarity and lipophilicity, and nature of the anionic moiety in the overall toxicity profile.

**Table 2:** Toxicity of some reported ILs to diverse life forms

IL	Biosystem	Comment on toxicity
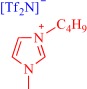	**Multicellular organisms**: *Daphnia magna*, *Pseudokirchneriella subcapitata*, and *Danio rerio*.	In vivo, IL is toxic to test organisms.^107^
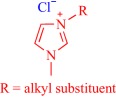	**Multicellular organism**: *Galleria mellonella*.	In vivo study suggested dependence of toxicity on length of alkyl chain.^108^
	**Nucleic acid**: Calf thymus DNA.	In vitro study showed helical conformation remains intact.^109^
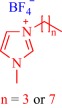	**Multicellular organism**: *Mytilus galloprovincialis*	In vivo tests showed lethal and nonlethal toxic effects.^110^
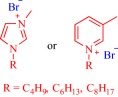	**Prokaryotic cell**: *Vibrio fischeri*, *Pseudomonas fluorescens*, *Saccharomyces cerevisiae*, *E. coli*, *S. aureus*, *B. subtilis*.	In vitro tests showed toxicity to all test organisms and in some cases more toxicity than common solvents such as benzene, chloroform, methanol.^101^
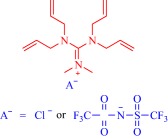	**Eukaryotic cell**: human colon carcinoma Caco‐2 cell.	In vitro tests reveal no toxic effects.^41^
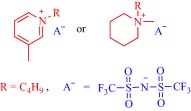	**Prokaryotic cell**: *V. fischeri*.	In vitro studies showed toxicity to test organism. Branching increases toxicity.^111^
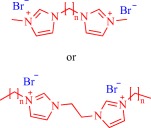	**Enzyme, Eukaryotic cells, and multicellular organism**: electric eel acetylcholinesterase, promyelocytic leukemia rat cells, *Scenedesmus vacuolatus*, *Daphnia magna*.	In vivo and in vitro studies indicated less toxic than monocationic ILs.^103^
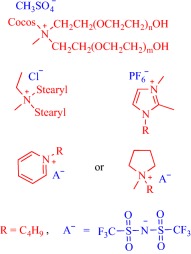	**Multicellular organism**: *Danio rerio*	In vivo, ILs are toxic to test organism with the ammonium‐based IL being more toxic than imidazolium, pyridinium, and pyrrolidinium‐IL as well as other common solvents.^112^

Judicious selection of the substituents on the cationic head group tunes toxicity as apparent from the effect of the dimethylamino group on the toxicity of pyridinium‐based ILs.^118^ 4‐Dimethylaminopyridinium‐based IL is more toxic to acetylcholinesterase^118^ and promyelotic leukemia rat cell line IPC‐81^120^ than its pyridinium congener. Perhaps, the electron‐donating dimethylamino group at the para‐position influences the electronic or solubility properties to tune the toxicity. Indeed, 4‐dimethylaminopyridinium is more lipophilic than pyridinium due to the substituent effect.^120^ The substituent can also interact with endogenous molecules to alter bioactivity. Such interaction may explain the lower inhibitory potential of alicyclic morpholinium cation on acetylcholinesterase compared to the aromatic cations including pyridinium, imidazolium, as well as alicyclic cations piperidinium, pyrollidinium.^118^ The ethereal oxygen in the comparatively less lipophilic morpholinium cation can hydrogen bond with endogenous water molecules, a phenomenon that will likely decrease interaction with endogenous hydrophobic moieties. With a more bioactive cationic head group such as 4‐dimethylaminopyridinium or quinolinium, toxicity depends on the cationic head group with negligible contribution from the side chain in contrast to less active groups such as imidazolium and pyridinium, where the side chain strongly modulates toxicity.^118^ Exemplarily, the toxicity of imidazolium, pyridinium, piperidinium, pyrollidinium, and morpholinium on the promyelotic leukemia rat cell line IPC‐81 is noticeably similar, suggesting lesser influence of the cationic head group.^120^ Also, the degree of branching of the substituent on the cationic head is pivotal to the extent of toxicity, but this effect operates differently in an aromatic and nonaromatic head group. Kurnia et al., for example, found that branching of the substituent decreases the toxicity of aromatic imidazolium and pyridinium‐based ILs toward *V. fisheri* but increases the toxicity of nonaromatic pyrrolidinium and piperidinium‐based ILs.^111^ The solubility of the aromatic imidazolium and pyridinium‐based ILs in water contributes to their toxicity toward *V. fisheri* since the EC_50_ values increase with aqueous solubility, a finding that agreed with the heuristic rule that hydrophobicity increases toxicity.^111^ But the rule is broken with the nonaromatic pyrrolidinium and piperidinium‐derived ILs, which lack a trend between EC_50_ and solubility, suggesting that factors outside hydrophobicity govern toxicity of this series.^111^

The need for benign ILs inspires the exploration of biomolecules like cholinium as cationic head groups. In some cases, the toxicity of bioinspired cholinium‐based ILs against many life forms is lower than that of the conventional ILs, for example imidazolium and pyridinium‐based ILs (Table 3). The lowest concentration of a cholinium‐based IL that inhibits the visible growth of *E. coli*, *S. Enteritidis*, *L. monocytogenes*, and *S. aureus* as well as inhibit the activity of acetylcholinesterase is an order of magnitude higher than that of an imidazolium‐based IL.^32^ Also, the low toxicity of cholinium‐based ILs is evident in the marginal changes they induce in the conformation of calf thymus DNA via weak interaction with the biomolecules, predominantly by electrostatic interaction through the grooves but with minor hydrophobic interactions.^39^ Unlike some imidazolium‐based chloride ILs that intercalate between adjacent DNA base pair,^121^ cholinium‐based ILs interactions are devoid of intercalative mechanisms since its nonplanar cationic head group may prefer peripheral electrostatic interactions. The cholinium‐based ILs interact solely using their cationic head with negligible contribution from the anion and side chain in contrast to some imidazolium‐based ILs, where electrostatic interactions from the charged head groups and hydrophobic interactions from the cation alkyl side chains drive size transition and conformational changes in DNA helical structure.^122^ Morpholinium‐based ILs, assumed to be relatively less toxic than imidazolium‐based ILs, exhibit a similar DNA binding mode as the cholinium class, using the nonplanar cationic head group to electrostatically bind to the minor groove of calf thymus DNA without disrupting the structural integrity of the DNA.^109^ It may be plausible to regard this series of ILs as benign to DNA since they weakly interact with DNA, albeit their strong affinity for DNA nucleobases, adenine, thymine, guanine, and cytosine, suggests otherwise.^39^

**Table 3:** Toxicity profile of some cholinium‐based ILs

Anion	Biosystem	Comment on toxicity
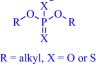	**Eukaryotic cell**: J774 murine macrophage	More toxic than NaCl.^37^
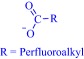	**Eukaryotic cell**: human lung fibroblast, human liver carcinoma cells, and human kidney fibroblast	Toxicity depends on the anion.^27^
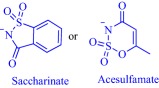	**Multicellular organism**: *Daphnia magna*	Less toxic than imidazolium and pyridinium ILs.^38^
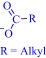	**Eukaryotic cell**: *Penicillium brevicompactum, P. glandicola, P. corylophilum, P. diversum*	Could be more toxic than ethanol with toxicity depending on length and branching of alkyl chain.^25^
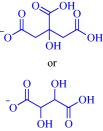	**Prokaryotic cell**: *V. fischeri*	More toxic than atrazine, benzene, 1‐butylpyridinium, 1‐butyl‐3‐methylimidazolium, and 1‐butyl‐1‐methylpyrrolidinium‐based ILs.^33^
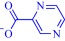	**Eukaryotic cell**: human colon carcinoma Caco‐2 and hepatocellular carcinoma HepG2 cells	No toxicity from cholinium cation. 34
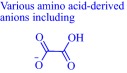	**Eukaryotic cell**: channel catfish ovary cells.	Very low toxicity with EC_50_ > 1,000 mg L^−1^.^44^

**Figure 8:**
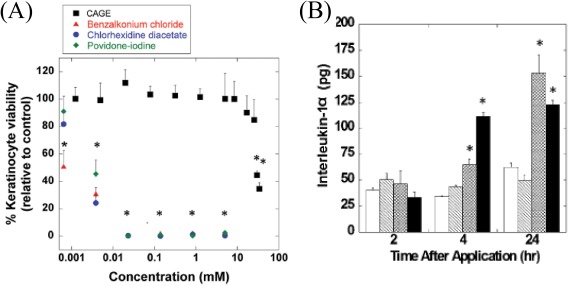
Choline geranate (CAGE) induces marginal skin irritation compared to traditional pharmaceutical excipients. (a) Viability of human keratinocyte cells after incubation with CAGE, and (b) secretion of interleukin‐1α, a biomarker for skin irritation (open bars, PBS negative control; hatched bars, CAGE; cross‐hatched bars, geranic acid; filled bars, %5 SDS positive control). Adapted from “Ionic Liquids as a Class of Materials for Transdermal Delivery and Pathogen Neutralization,” by M. Zakrewsky et al., 2014, Proceedings of National Academy of Sciences of the United States of America, 111, pp. 13313–13318

Our group investigated the biocompatibility of a cholinium‐based IL, CAGE, toward a primary adult keratinocyte cell line and found it more benign than the precursors, choline, and geranic acid.^31^ Specifically, CAGE causes less skin irritation than its precursors as evidenced by the marginal change in the integrated peak area between 1,650 and 1,660 cm^−1^ in the Fourier transform infrared spectroscopy (FTIR), an area that corresponds to α‐helix content of the SC. Again, the insignificant amount of the pro‐inflammatory cytokine, interleukin‐1α (IL‐1α), secreted from reconstructed human epidermal cultures in the presence of CAGE (Figure 8) confirmed nonirritancy. It is presumed that irritants provoke skin irritation via surfactant‐triggered disruption of keratinocyte to release IL‐1α, reactive oxygen species activation of transcription factors to promote secretion of IL‐1α, or fluidization of keratinocyte membrane liposomal bilayers to initiate irritation signal transduction.^123^ As CAGE induces marginal irritation, it is logical to assume that it evades these mechanistic pathways, especially that involving the production of IL‐1α. Regardless, CAGE biocompatibility is selective, being toxic to a wide range of microorganisms including fungi, viruses, and bacteria but relatively benign to a human epidermal keratinocytes cell line. The benign behavior of CAGE was attributed to ion‐pairing between the cationic choline and the anionic geranate, a phenomenon that shield the ions from interacting with and disrupting keratinocyte membrane to provoke irritation.^29^ A recent study from our group confirms that CAGE extracts lipids from the SC as revealed by FTIR data where the integrated peak areas of the CH_2_ asymmetric and symmetric stretching of SC lipid at 2,920 and 2,850 cm^−1^ decrease after incubation with CAGE.^30^ The findings suggest that ILs, such as CAGE, are promising biocompatible technologies to enhance transdermal drug delivery.

The concept of a generically benign cholinium‐based IL is speculative as toxicity depends, as with other ILs, on the nature of anion, side chain, and biosystem. Indeed, depending on the anion and the biosystem, some cholinium‐based ILs could be toxic (Table 3).^31^ The IC_50_ of choline oleate on normal human bronchial epithelial cells is in the low millimolar range (IC_50_ = 0.034 mM) but overall, the cholinium‐based ILs are by an order of magnitude less toxic against this cell line than tetraalkylphosphonium geranate.^31^ Further, cholinium bitartrate is more toxic (EC_50_ = 38 mg L^−1^) toward the *V. fischeri*, than 1‐butylpyridinium chloride (EC_50_ = 258 mg L^−1^), 1‐butyl‐3‐methylimidazolium chloride (EC_50_ = 548 mg L^−1^), 1‐butyl‐1‐methylpyrrolidinium chloride (EC_50_ = 7,980 mg L^−1^), and dimethylethylbutylammonium chloride (EC_50_ = 3,314 mg L^−1^).^33^ Also, an in vivo study showed that cholinium‐based deep eutectic solvents are more toxic than their constituent compounds to ICR mice.^124^ This study revealed that the molar ratio of the constituents is central to the toxicity since a 1:2 mixture of choline chloride and urea is less toxic than the 1:3 mixture.^124^ Taken together, the toxicity of ILs depends on a complex interplay of structural features, concentration, and the investigated biosystem and so, through rational control of these parameters, it is feasible to design biocompatible ILs.

## 2 CONCLUSION AND RECOMMENDATIONS

The objective of this review is to summarize and further motivate biomedical application‐driven exploration of ILs. By exploiting the rich structural diversity of the ions and the flexibility to facilely pair these ions, the properties of ILs can be tailored to meet a wide range of biomedical needs. Advances in this direction have shown the ability of ILs to enhance drug permeation through physiological barriers, promote the dissolution of poorly soluble drugs, and provide a strategy to design API‐ILs. The results reported have been very encouraging, with enhanced bioavailability and efficacy the hallmark of this IL‐strategy. Also, ILs are emerging as technology to combat infectious diseases. It is now established that ILs are potent and broad‐spectrum antimicrobial agents with activities that surpass many conventional antimicrobial agents. The excitement generated by this application‐driven research has attracted organometallic chemists to incorporate transition metals into ILs with the expectation of accessing exotic properties such as luminescence, redox activity, and magnetism. Although the field of metal‐containing ILs is at the early stage of development, it has shown promise in biosensing application as exemplified by the use of a Co‐containing IL to sense glucose. Therefore, ILs should be considered as a promising technology to address many challenges such as drug bioavailability, control of infectious diseases, and biosensing in the biomedical field. We envision that the emerging field of metal‐containing ILs could provide new opportunities, for instance in bioimaging given the optical, magnetic, and radioactive properties of metals.

Depending on concentration and structural features, the toxicity profile of ILs may vary, but by rational design, the toxicity can be mitigated. This understanding, albeit encouraging, is over‐simplified given that toxicity also depends on the biosystem, which typically co‐exists with other life forms in an ecosystem. Hence, it is crucial that susceptibility of the diverse life forms in an ecosystem be considered during the design stage and investigated in vivo using mammalian models. Toward this, we recommend designing ILs with target specificity, as that could limit access to nontargeted sites and life forms. The antimicrobial function of cationic metal‐containing molecules is now recognized and should provide the impetus to incorporate metals into ILs to tackle pathogens. Also, it is demonstrated that the presence of a metal center alters the solubility behavior of molecules, a finding that should form a foundation for the exploration of metal‐containing ILs in the design of API‐ILs. In conclusion, ILs present a promising and under‐explored therapeutic opportunity with immense potentials.
